# Discrepancies in the spiking threshold and frequency sensitivity of nocturnal moths explainable by biases in the canonical auditory stimulation method

**DOI:** 10.1098/rsos.172404

**Published:** 2018-04-11

**Authors:** Herve Thevenon, Gerit Pfuhl

**Affiliations:** Department of Psychology, University of Tromsø, N-9037 Tromsø, Norway

**Keywords:** tympanal organ, auditory system, evolutionary arms race, invertebrate acoustics

## Abstract

The auditory stimulation method used in experiments on moth A cell(s) is generally believed to be adequate to characterize the encoding of bat echolocation signals. The stimulation method hosts, though, several biases. Their compounded effects can explain a range of discrepancies between the reported electrophysiological recordings and significantly alter the current interpretation. To test the hypothesis that the bias may significantly alter our current understanding of the moth's auditory transducer characteristics, papers using the same auditory stimulation method and reporting on either spiking threshold or spiking activity of the moth's A cells were analysed. The consistency of the reported data was assessed. A range of corrections issued from best practices and theoretical background were applied to the data in an attempt to re-interpret the data. We found that it is not possible to apply *a posteriori* corrections to all data and bias. However the corrected data indicate that the A cell's spiking may (i) be independent of the repetition rate, (ii) be maximum when detecting long and low-intensity pulses and (iii) steadily reduce as the bat closes on the moth. These observations raise the possibility that a fixed action pattern drives the moths' erratic evasive manoeuvres until the final moment. In-depth investigations of the potential bias also suggest that the auditory transducer's response may be constant for a larger frequency range than thought so far, and provide clues to explain the negative taxis in response to the searching bats' calls detection.

## 1. Introduction

It has been observed that moths apply a range of evasive manoeuvres that appear to depend on the distance of the moth to its main predator: the bat [1]. The likelihood to successfully escape an attack when moths are released 6 m away from a group of foraging bats is 45% [2], independent of their family. This suggests the outcome of the moth's manoeuvres is not driven by chance. Dissections have shown that moths of the Notodontidae family have a single neuron in their tympanal organ—the A cell—[3] that is responsible for the transduction of ultrasounds typically emitted by their foraging predators. Other families equipped with up to four A cells per ear do not display a significant difference in behaviour or escape rate [1].

**Figure 1.**
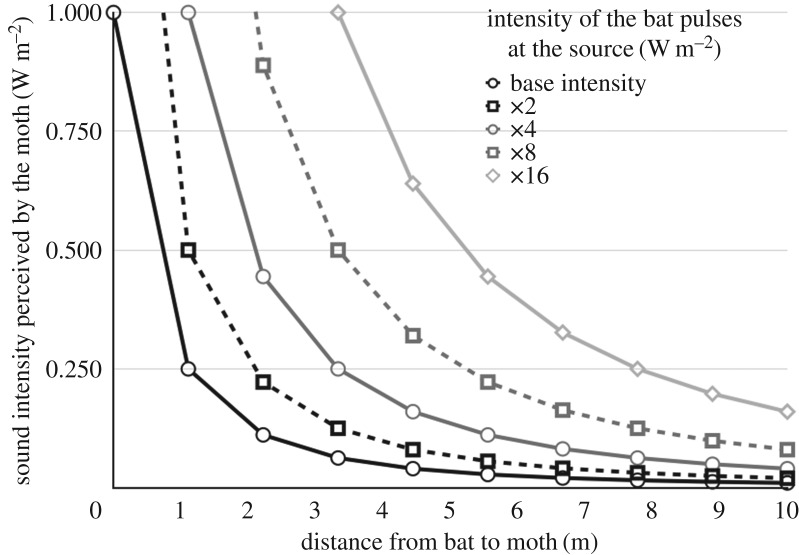
The intensity of the signal received by the moth may have been produced by an infinity of (intensity at source, distance) combinations.

In anthropomorphic terms, the moth seems to evaluate its distance to the bat by using some characteristics of the bat's own echolocation pulses. The pulses' intensity is an intuitive parameter for this evaluation and its effect on A cell spiking has been studied for more than 50 years. It is well known that the intensity of sound waves decreases with the square of the distance. Therefore, the instant evaluation of the intensity of the sounds it receives does not allow the moth to decide whether its predator is far or close. Figure 1 illustrates that from a moth's perspective a signal received with a given intensity may have been produced by many combinations of the intensity of the source and distance to the source.

In addition, the ultrasonic pulses of the foraging bat vary during the chase: usually, the pulses’ intensity and duration decrease with the distance to the prey while pulses are emitted more frequently [4]. Indeed, this observation makes the moth's intensity-triggered evasive behaviour even more unlikely. It also opens a host of possibilities for the potential parameters involved in the moth's calculations beside pulse intensity: pulse duration, pulse repetition rate, pulse carrier frequency and integration over time. The actual combination remains to be determined in order to understand how the complex survival behaviours of the moth are elicited with—for some of them—only one neuron bridging the environment and the nervous system [5].

Since 1957, many experiments have been conducted to characterize the spiking threshold and activity of the nocturnal moths' A cells. Since the mid-1980s, the same investigation method has been applied on different families and species of moths. This investigation method consists of a moth tethered for extracellular recordings of one of its tympanal nerves whose related ear is stimulated by a sound source emitting ultrasonic pulses. The intensity of the sound received by the moth is controlled by the experimenter by reading the measurement made by a sound pressure level (SPL) meter connected to a microphone.

This method has intrinsic biases that we will introduce as required through the course of this paper. The main concern is with the measurement of the sound intensity. What is actually measured via the equipment used in the method is sound pressure, not sound intensity. The rigorous definition of sound intensity is the ‘vector quantity equal to the product of the sound pressure and the associated fluid particle velocity vector’ [6]. It is measured in Watt/m^2^ and describes the flow of energies (e.g. kinetic, potential, heat) through a surface. Sound pressure is a component of sound intensity that is measured in Pascal. Sound pressure may be used as a proxy for sound intensity under specific conditions. As will be seen later, these conditions were not met during the experiments we analysed. This is important because the sound pressure reported is universally used throughout and across experiments using the same method, in order to establish comparisons and draw conclusions regarding the characteristics of the moth's ear as an energy detector [7,8].

In the absence of technology available to measure sound intensity above 20 kHz [9], microphones remain the most practical tools to evaluate sound intensity in the frequency range that matters to many moths species: 20–100 kHz.

**Figure 2.**
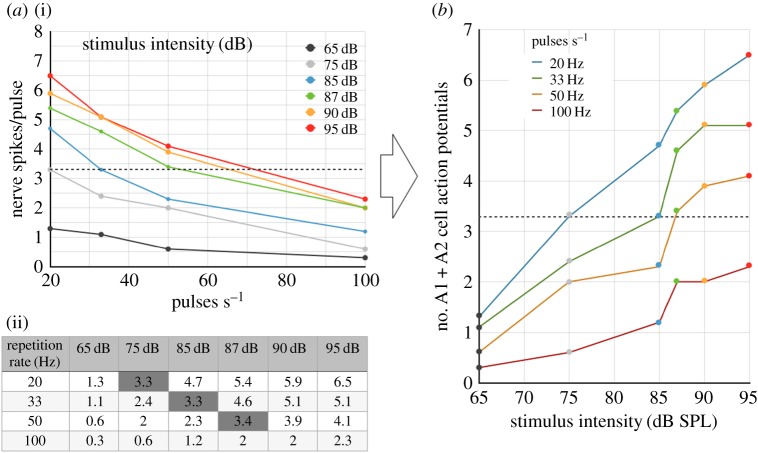
According to Fullard [10], the higher the repetition rate, the more intensity is required to elicit a given spiking activity per stimulus. (*a*)(i) Graph modified from Fullard. (ii) Data tabulated. (*b*) Graph transposed for easier comparison with figure 3. The horizontal dotted lines indicate an equivalent level of spiking arbitrarily set to 3.3 spikes per pulse.

**Figure 3.**
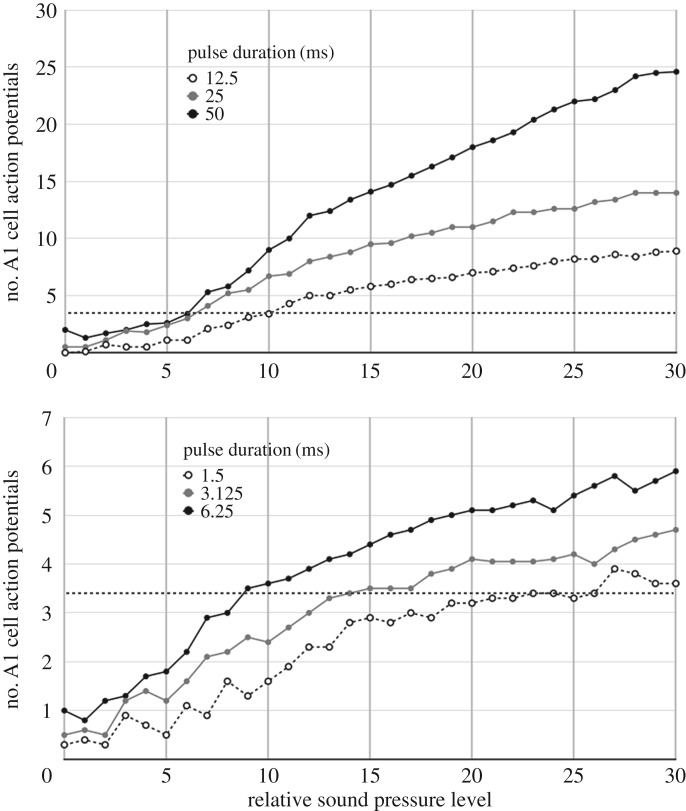
According to Waters [11], the longer the pulse duration, the less intensity is required to elicit a given spiking activity per stimulus. Repetition rate is 1 Hz. As in figure 2, the dotted lines indicate an equivalent level of spiking arbitrarily set to 3.3 spikes per pulse. The relative SPL uses the first spike of the A cell as zero and all other measurements are relative to this animal baseline.

The significant variations in the data reported by a series of experiments selected for their methodological similarities were the reasons for us to look into how the auditory stimulation method was implemented. For example, Fullard's data [10] show that the SPL required to elicit a given spiking activity increases as the pulse repetition rate increases for pulses of equal duration. Conversely, Waters' data [11] show that the SPL required to elicit a given spiking activity decreases as the pulse duration increases at a constant repetition rate. The data from both studies are reported in figures 2 and 3. The energy received by the moth's ear (over a given period of time) is directly proportional to the pulse duration and repetition rate. If the energy detector hypothesis holds in a consistent way for both Fullard and Waters, it means that pulse duration is not a variable, only SPL. However, the pulse duration is a correlated variable of SPL for both Fullard and Waters data, hence both conclusions and the energy detector hypothesis cannot be true simultaneously.

Here, we provide three results that emerged after scrutinizing known biases in the stimulation method, e.g. shortcomings of the SPL meter, and applying standard acoustic ‘corrections’. Briefly, experimental data collections obtained at fixed frequency may be corrected by inferring the actual SPL received by the microphones. A new conclusion arising from a dataset published by Waters is that the A cell firing starts when the bat is in search mode and far from the moth, and the firing is maximum. Furthermore, from one of Fullard's papers we conclude that the firing is independent of the echolocation repetition rate. Taken together, these results suggest the pulse duration is the key to A cell firing. Next, we provide our analysis steps which rely heavily on acoustics and theory of operation of the 1960s SPL meters. This background allows us to devise a method to infer the actual incident SPL. We also borrow from sound-level measurement best practices as developed in aeronautics. Finally, when analysing the properties of the sound stimulation method across all the papers reviewed, we formulate an alternative approach to minimize biases.

## 2. Methods and results

### 2.1. Literature selection

First, we searched for papers containing the keywords ‘moth’ or ‘Lepidoptera’ and ‘auditory’ or ‘acoustic’ in Scopus and ISI Web of Science, and checked all the references for additional materials. In order to be selected, the papers had to report original data for one or both of the following experiments conducted on the nocturnal moths: (i) spiking frequency as a function of carrier frequency, pulse duration and sound pressure level, and (ii) spiking threshold as a function of sound pressure level, pulse frequency and pulse duration. This is a natural choice when one considers that the spiking frequency and threshold are the most basic characteristics that may be studied in this context, and therefore the most studied. Such results can only be compared if the experimental conditions and the auditory stimulation method are well described. Consequently, the selected papers provide specific details about the auditory stimulation method applied: pulse duration, pulse shape, pulse repetition rate and frequencies being used. As all the papers presented the data as graphs, the selection process discarded those papers whose data could not be tabulated, typically because units or scale were missing. In order to assess the bias specific to experiments involving ultrasounds, the papers retained include most of the following details: the make and model of the sound source, microphone and SPL meter, distance and incidence of the sound source to the preparation, and incidence of the microphone with regards to the sound source. The final selection consists of 12 papers published between 1983 and 2011 that encompass a range of 10 species and six families. Seven papers [11–16] reported on spiking activity, specifically the number of spikes obtained with stimuli varying in sound pressure level and pulse duration, at a fixed frequency (electronic supplementary material, table S1a). Nine papers [15–22] reported on spiking threshold, specifically the pulse frequency and sound pressure level required for a stimuli of a given pulse duration to elicit spiking threshold (electronic supplementary material, table S1b).

However, as will become clear below, for correcting the systematic bias of the SPL meter, we could only use two studies that used similar experimental conditions on two species belonging to the same superfamily [10,22]. For taking into account the incidence error in microphones, only one study provided enough details [18]. The remaining papers had insufficient details to carry out the corrections (see the electronic supplementary material, tables S1a and S1b). Nevertheless, given similar peripheral processing across insect taxa [23], differences between moth species should be of minor importance with regards to our focus.

### 2.2. Systematic bias of SPL meters

Late 1960s SPL meters were not suitable to control the intensity of a pulsed sound source. Essentially, these SPL meters were galvanometers enhanced with a time-weighting damping circuit—applied to the signal received from the microphone used as input—to facilitate the readings. The effect of the analogue electronic circuit used for this damping can be expressed in mathematical terms [24] as the following equation:

2.1 $$\left\langle p_{A}^{2}(t)\right\rangle =\frac{1}{\tau }\int_{-\infty }^{t}p_{A}^{2}(\xi )\;{\text{e}}^{-(t-\xi )/\tau }\partial \xi ,$$

where *p*_A_ is the sound pressure (in Pascal), *τ* is the damping time constant (1 s for the S setting and 0.125 s for the F setting), *ξ* is the integration variable, and *t* is the time of the observation. The older the signal, the less significant.

The SPL meter reports dB SPL. dB SPL is obtained by dividing the square of the measured sound pressure by the square of sound pressure of reference (20 μPa). In order to compute equation (2.1), we expressed it as a Riemann sum shown as the numerator in equation (2.2). This expression factors out the constants found in equation (2.1), and by convention *ξ* is replaced by *x*. Equation (2.2) was coded to calculate and graph the movements of the SPL meter's pointer over time as the meter receives pulses.

2.2 $$\text{d}B_{t}=\text{lo}{\text{g}}_{10}\left(\frac{(e^{-t/\tau }\Delta x\text{/}\tau )\sum_{x=-\infty }^{t}{\text{e}}^{x/\tau }p{\left(x\right)}^{2}}{p_{\mathrm{ref}}^{2}}\right).$$

In practice, the −∞ boundary in (2.2) must be replaced by a more computable value ‘*z*’. Drawing from Marsh's considerations [24] we found that *z* = −5 ms is computationally efficient and representative of –∞ for any of the trapezoidal signals used in the remainder of the paper.

**Figure 4.**
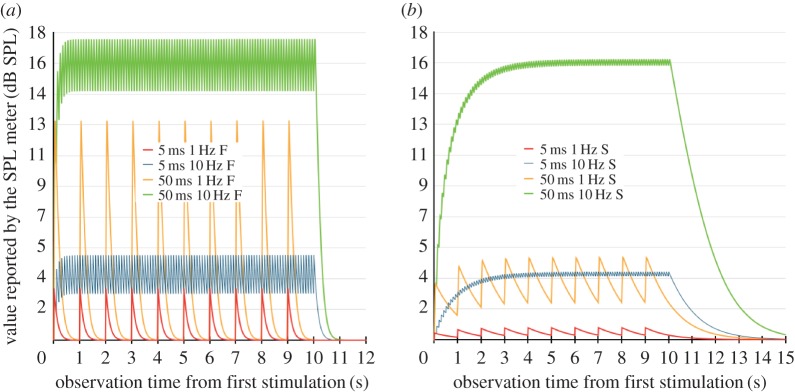
Graph of the SPL meter's pointer movements for 5 ms and 50 ms signals, repeated once or 10 times per second, evaluated using the (*a*) F and (*b*) S settings.

Figure 4 presents an example of these calculations for pulses whose duration is either 5 or 50 ms, and whose repetition rate is either 1 or 10 Hz. The intensity of the signals is arbitrarily set to 60 µPa—i.e. three times the SPL of reference, or 9.5 dB SPL—for all eight scenarios. The figure clearly illustrates the difference between the S and F settings. On the F setting, the graph indicates that the pointer jets across the display with a speed that makes the readings subjective. Since the SPL meter setting was never documented in our selection of papers, we assume that the S setting was used, and we used this setting for all the results presented in this paper.

On this basis, it is possible to infer the actual sound pressure level that would have been returned by an SPL meter complying with equation (2.1) for a series of measurements that have in common three of the four following parameters: (i) pulse shape and duration, (ii) pulse carrier frequency, (iii) pulse repetition rate, and (iv) measured dB SPL. The method consists of four steps: (1) We first simulate the response of the SPL meter for the kind of pulses used in the experiment and for a series of emitted sound pressure levels, then (2) we find a suitable regression that allows to convert the RMS dB SPL readings into the sound pressure level that was received by the SPL meter's microphone. Next, (3) we replace the RMS dB SPL value as reported in the papers by the corresponding dB SPL value obtained via the regression formula, and finally (4) we graph the new dataset.

**Figure 5.**
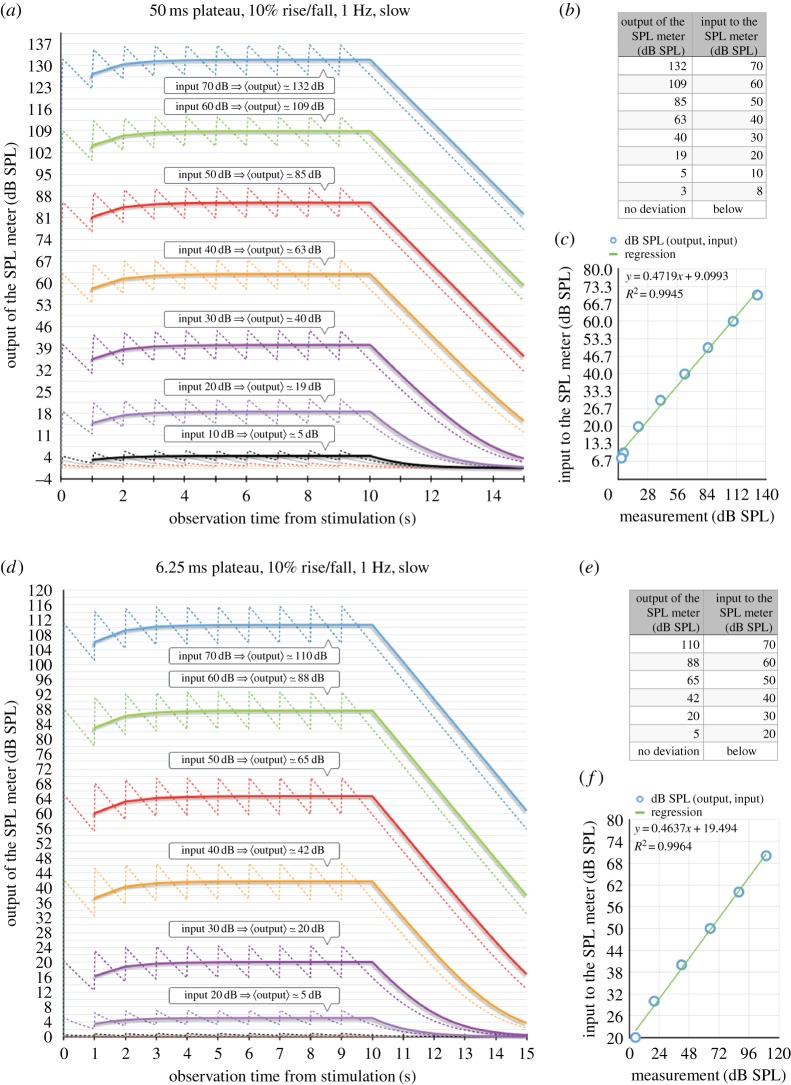
Illustration of steps 1 and 2. Step 1: (*a*) and (*d*) For each plateau duration, sample the emitted sound pressure space and derive the RMS dB SPL value calculated by the SPL meter according to equation (2.1), (*b*) and (*e*) provides the conversion, (*c*) and (*f*) Step 2: Find a suitable regression that converts RMS readings to the actual emitted sound pressure level.

**Figure 6.**
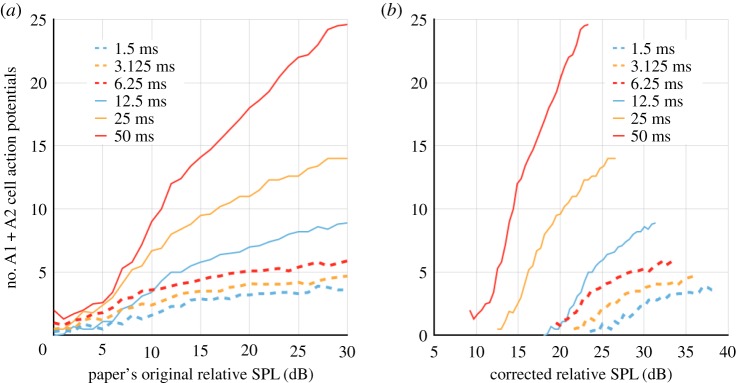
Effect of the correction on Waters' data [11]. (*a*) Waters’ original results on the same graph. (*b*) The same data without the systematic error induced by the damping circuit of the SPL meter.

**Figure 7.**
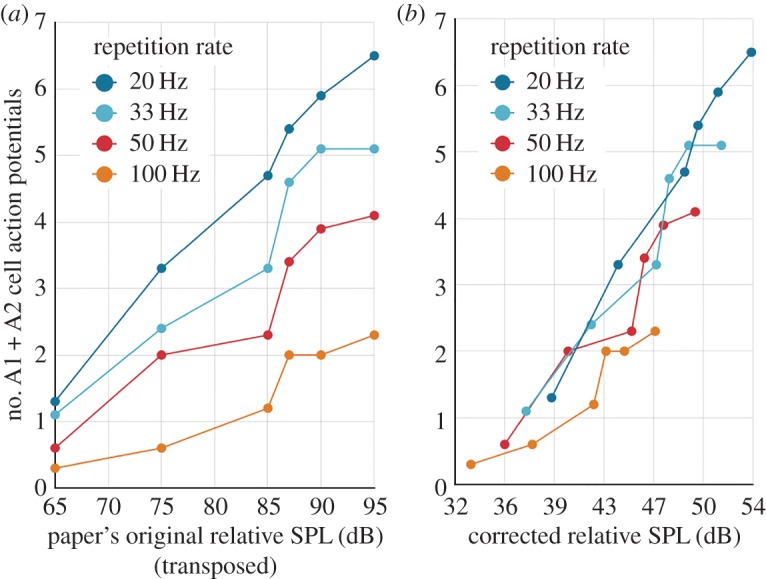
Effect of the correction on Fullard's data [10]. (*a*) Fullard's original data. (*b*) The same data without the systematic error induced by the damping circuit of the SPL meter.

We sampled the response of the mathematical SPL meter for signals of different sound pressures for each different plateau duration for Water's [11] experiment. For each plateau duration, we extracted a regression rule that allowed to infer the emitted sound pressure level that matches the experimenter's reading. Figure 5 shows two examples for plateau durations equal to 50 ms and 6.25 ms respectively. Figures 6 and 7 are the outcome of step 3.

### 2.3. Microphone incidence error

**Figure 8.**
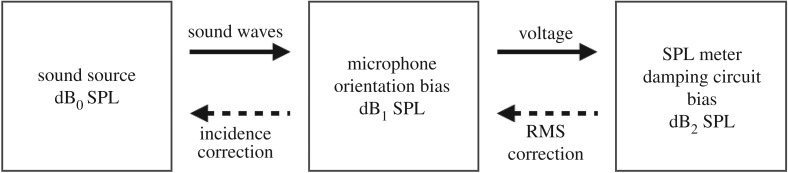
Compounded errors and order of corrections.

Microphones are sensitive to the incidence of the sound waves on the membrane and the grid, and this sensitivity varies with the signals' carrier frequency. The linearity of the microphone's response is guaranteed for normal incidence and grid on. Therefore, if the microphone's incidence deviates from 0°, the correction consists of two steps. First one must find the actuator response for the given frequency by removing the 0° correction for normal incidence and then add up the correction related to the reported degrees incidence, i.e. in Madsen and Miller [18] this was 150°. Since an SPL meter was involved to report the sound pressure level, the same correction method applied for Fullard's and Waters’ data should indeed be applied on Madsen's and Miller's data. But which correction should be applied first? The diagram in figure 8 explains the reasoning: the SPL meter's displayed measure dB_2_ is based on the microphone's output dB_1_, therefore dB_2_ can be expressed as dB_2_ = *Ε*_d_(dB_1_) = *E*_d_(*E_i_*(dB_0_)) with *E*_d_ and *E*_i_ the respective damping and incidence errors induced by the equipment.

Accordingly, we simulated the SPL response to a trapezoidal signal of 10 ms including a 0.5 ms rise/fall time repeated 10 times per second emitted with different sound pressure levels and a linear regression describing the transformation dB_1_ = T(dB_2_) derived. Subsequently, a series of incidence corrections were applied by using the principle presented above and the manufacturer's specifications.

### 2.4. Application to experimental data

The correction process does not attempt to derive absolute measurements from relative measurements. That is not possible. However, what is possible is to correct the slope of the dB SPL reported within the context of each stimulus {frequency, repetition rate}. For two datasets [10,11] both using fixed frequency stimulation, we could gauge the actual SPL received by the microphones. These two papers report data about *Agrotis segetum*, from the Noctuidae family and *Cycnia tenera*, a member of the Erebidae family—both belonging to the Noctuiodea super-family. Both species have two A cells located on the metathorax.

In the first instance, we re-analysed data reported by Waters [11]. We refer to the experiment pertaining to the spiking activity of the A1 cell given different pulse duration and the SPL as measured by the SPL meter. The sound stimulation consisted of trapezoidal signals emitted with a constant SPL with a rise/fall duration equal to 10% of a series of plateau durations. The original result stems from two series of experiments (the three shortest durations and the three longest durations).

Waters’ data corrected for the SPL meter's pulse duration-related bias suggest that the A1 cell's firing rate would be higher when the bat is far away (longer pulses and lower SPL), and would become lower as the bat gets closer (shorter pulses and higher SPL). Waters originally split his experiment into two groups—the shortest plateau durations in one group, and the longest in the other—and reported SPL as relative to the spiking threshold obtained for the shortest duration in each group. Therefore, these relative SPLs differ by an unknown offset that is the likely cause for the two groups' original data to overlap when superimposed. The corrected datasets suggest that more SPL was required to obtain the baseline spiking threshold in the lowest durations group than in the highest durations group, and therefore the lowest durations group's data should have slightly higher SPL than shown (i.e. should be shifted to the right). With or without this caveat, this result departs from the general view developed through the papers selected. Because inputs with same SPL and different pulse durations produce a different spiking activity, we suggest that the pulse duration is as significant to the auditory transducer's output as the received SPL.

Fullard [10] investigated the effect of different repetition rates in *Cycnia tenera.* Since the design of all SPL meters used in this field of research has the same inherent systematic error, we corrected Fullard's data with the same method.

Fullard concluded that the repetition rate was ‘a cue for defensive behaviour’. The corrected data tell the opposite: the spiking frequency is not correlated to the pulse repetition rate when the bias of the SPL meter is taken into account. A linear regression can be applied to the corrected data and reasonably fits the sample of points independently of their respective repetition rate (electronic supplementary material, figure S1). The residuals did not comply with the Gaussian distribution to back this conclusion, but this may be explained by the small number of samples and their uneven distribution over the SPL new interval.

Secondly, experimental data collections obtained while varying frequency are biased by the microphone orientation. *A posteriori* corrections cannot be applied. However, Madsen and Miller's data and most detailed method [18] suggest that *Mamestra brassicae* (Noctuidae) are insensitive to variations of frequencies in the range 20 kHz–60 kHz.

**Figure 9.**
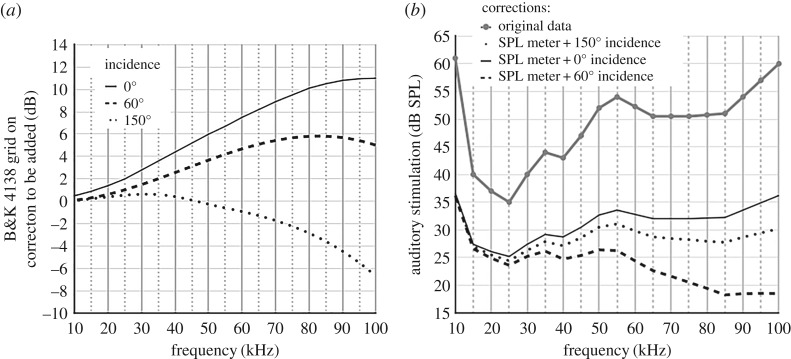
Influence of the microphone orientation on the SPL measurement. (*a*) Correction of incidence using B&K corrections chart for the 4138 microphone with grid on. (*b*) The graph shows the original data, the SPL meter bias correction alone, and several microphone incidence corrections compounded with the SPL meter bias correction.

The characterization of the spiking threshold of the A cells was performed across a range of carrier frequencies varying from 20 to 100 kHz. Madsen and Miller reported data on *Barathra brassicae* (Noctuidae) while using a B&K4138 microphone with a grid on. The moth was placed with a 150° angle to the sound source and we can therefore assume that the microphone would have been oriented similarly. Accordingly, we first applied an SPL meter correction and followed with a series of incidence corrections. The result is presented in figure 9.

This example shows the importance of the microphone incidence bias when compounded with the SPL meter bias. The correction is frequency dependent and can reach 70 dB. The removal of the grid requires to apply similar corrections and does not reduce the biases' impact. None of the papers investigated allowed to apply incidence corrections retrospectively, either because the data are missing, or because the interpretation is ambiguous (electronic supplementary material, tables S2a and S2b).

Thirdly, without meeting free-field conditions and using proper sound sources, all the results are likely to be biased by interferences patterns and distorted signals, whose impacts cannot be asserted. This collection of biases inherent to the auditory stimulation method leads to suggest an alternative sound stimulation method.

### 2.5. Minimizing the biases

Free-field conditions are met when the space around the measuring microphone is exempt from reflected sound waves (including standing waves) and interference. This condition may be met if (i) there is only one sound source that can stimulate the moth, and (ii) the distance between the sound source and any obstacle—including the preparation—is larger than the distance travelled by the sound wave during the pulse duration.

The first condition can be met by shielding the experimentation area against external sound sources in the frequency range 20–100 kHz. Anechoic chambers have reportedly been used for this purpose in several experimental set-ups. Failure to do so means that external sound sources may interfere with the auditory stimulation and contribute to generate spiking for a range of frequencies outside of the stimulation protocol.

Given the second condition and the average propagation speed of sounds in the air of 340 m s^−1^, reflections will definitely be avoided if 5 ms pulses are given a 1.65 m clearance and if 30 ms pulses are given 10 m, in every direction. The minimum distance should then be increased accordingly for repeated pulses if the repetition rate is likely to generate interferences at the measurement distance. This places practical constraints on the dimensions of the experimental set-up that anechoic chambers rated for 20–100 kHz range can reduce.

Beside practical considerations, one should also consider the theory. To make sure that the measurements are significant, acousticians refer to the far-field or Fraunhofer distance: a distance where the diffraction of the sound waves created by the sound source itself becomes negligible enough to not create interferences at the distance of measurement. The Fraunhofer distance is defined as twice the sound source's largest dimension squared divided by the wavelength of the sound wave. Table 1 shows all the experiments placed the preparation too close to the sound source to ensure correct measurements. The free field is defined as a subset of the far field where there is no interference. The standard free-field test shows that the intensity of the sound decreases by 6 dB as the distance to the sound source doubles.

**Table 1.** Inventory of the sound sources used in the selected experiments. The table reports on physical dimensions, frequency rating, number of times used in the experiments reporting on spiking activity and threshold.

sound sources: manufacturer and model (or paper when custom made)	Technics TH400B	KEF T27	Avisoft Scanspeak	PH10	Madsen & Miller [18]	Skals & Surlykke [20]
number of spiking activity papers	3	1	0	1	0	0
number of spiking threshold papers	4	0	2	0	1	1
rated frequency spectrum (kHz)	0–20	0–20	0–100	n.a	n.a	n.a
largest dimension (mm)^a^	70	19	32	5	15	60
distance to preparation (mm)	300 or 400	100	300	4	40	n.a
Fraunhofer distance (mm) @100 kHz	2882	212	602	15	132	2118

^a^Speakers’ dimension from papers, manufacturer's documentation or evaluated from picture (TH400B).

### 2.6. Basis for a new auditory stimulation method

Auditory stimulations require pre-calibrated sound sources designed to operate in the range 10–100 kHz, as a whole or for a specific frequency. The sound sources should present a low level of linear and nonlinear distortions and be accompanied by manufacturer supplied free-field correction charts. Pre-calibration must be achieved for the whole sound source system, including the air interface and its pulse/intensity driver.

The sound source and the preparation should be shielded from any other sound source, including any electronic equipment used for the purpose of the experiment. Therefore, the sound source driver should be connected to the sound source by a cable long enough to allow for the driver to be outside the shielding enclosure. Thanks to the preliminary calibration of the sound source and its pulse/intensity driver, there will be no requirement to insert a microphone within the sound field when the preparation is in the free-field space. The use of a microphone in the shielding enclosure should be limited to test for free-field conditions and confirm the sound source calibration and integrity before and after the experiment.

The sound sources may be mono-frequency air transducers. Their small size and diffraction angle weigh positively on the space required to achieve free-field conditions. The sound source and driver should be designed to travel easily between laboratories and prevent tampering. Ideally, the sound source system can be pre-loaded with a test plan (typically a text file on a removable media) that would allow to stimulate the moth automatically and record the electrophysiology offline. Given the stimulation frequency, the encoded electrophysiological data can be paired with the stimuli by a third party, thus allowing for double-blind experiments.

Since there is virtually no sound intensity probe that operates above 10 kHz, the experiments require free-field conditions. This may be achieved with a large cylindrical chamber that will enclose the sound source and the preparation only, and shield them from any external sound source. Roeder & Treat [25] should be noted for presenting a similar set-up, that seemed to borrow from waveguide theory. We suggest to make the cylinder long and wide enough to minimize internal reflections. This can be planned by using the sound source's manufacturer specifications. Length will mainly depend on the size of the sound source. It should be several multiples of the most conservative Fraunhofer distance, so as to allow for testing free-field conditions within the cylinder. The sound source should protrude slightly from one extremity of the cylinder. The other extremity of the cylinder will be prepared with anechoic structure and materials suitable to absorb sound waves in the range 10–100 kHz. If such material cannot be found or is too expensive to source, the shield will need to be extended so as to prevent reflections from interfering with the incoming pulses from the source in the area of the preparation.

The preparation should be installed in the midline of the cylinder using fittings whose size will not diffract the wave plane and create unwanted interferences. A thin gauge (sub-wavelength diameter) taught cable may be used for this purpose. Electrode wires should run longitudinally so as to avoid reflections that could interfere around the preparation. Whether the set-up is adequate can be determined using the canonical free-field test: 6 dB decrease as the distance doubles.

## 3. Discussion

In this paper, we have shown that the conditions required to perform accurate acoustic measurements of the sound pressure levels required to characterize the auditory transducer of nocturnal moths are never met, and therefore do not allow to transpose SPL (dB) to proper sound intensity (W m^−2^). As a result, none of the experiments reviewed could possibly answer the question: Is the moth's ear an energy detector? We have seen that sound pressure levels were controlled via a method flawed by several important biases, and that the reported results could contradict one another for the same species. As a counter measure, we developed a corrective method that allowed to reconcile discrepant results when each experiment is based on a single carrier frequency that may be different between experiments. We also found that we cannot apply *a posteriori* corrections on experiments that perform sound pressure measurements over a range of frequencies, because of three main factors: the actual incidence of the microphone to the sound source is never described in non-ambiguous terms, the distance between the sound source and the preparation is insufficient to prevent interferences in the area of the microphone, and some sound sources are basically inadequate for the task (the TH400B's diffusion plate effectively creates two distinct sound sources).

The results obtained after applying the corrective method developed in this paper suggest a re-evaluation on three levels. (i) A new auditory stimulation method is required. (ii) Based on Fullard's experiments and the novel corrective method presented in this paper, the A cell's spiking activity seems to be independent of the pulse repetition rate. (iii) Corrections of Waters' results suggest that the pulse duration is the key trigger to evoke spiking.

If the new results produced using Fullard's and Waters’ published data stand the test of experiment, new iterations of the experiments conducted by Madsen & Miller [18] should be conducted to determine whether the moths enter any fixed action patterns after reception of a bat searching call and sustained stimulation with approaching calls. In this work, they noted that ‘when the flight oscillator is running auditory stimuli can modulate neuronal responses in different ways depending on some unknown state of the nervous system’. From another perspective, the body of experimental data suggests that moths detect searching bats before bats can detect them. As a consequence, experiments in the vein of those by Acharya and Fenton [2] pertaining to studying the attack success rate of preying bats on moths released in the vicinity of their foraging area should be extended to larger release distances, from 10 to 25 m away from the foraging area. The rationale is that when released within a 6 m radius of search calls, without being shielded from those searching calls, the moth's nervous system may have already modified its internal state. As a result, the current results may not reflect the benefit for the moth to detect a crowd of foraging bats before being detected, and subsequently avoid confrontation.

Fullard's interpretation that the A cell encodes the pulse repetition rate did explain that some moth species exploit the pulse repetition rate to distinguish between bat calls and mating calls [26]. Our interpretation suggests that the exploitation of the pulse rate may take place at the interneuron level [27–29].

## Supplementary Material

Figure S1

## Supplementary Material

Table S1

## Supplementary Material

Table S2

## Data accessibility

Calculations can be found in the Dryad Digital Repository at https://doi.org/10.5061/dryad.4b60f [30].
