# Supplementary material for: Discrepancies in the spiking threshold and frequency sensitivity of nocturnal moths explainable by biases in the canonical auditory stimulation method
Source: R Soc Open Sci. 2018 Apr 11;5(4):172404. doi: 10.1098/rsos.172404 (PMC5936950; doi:10.1098/rsos.172404)
Supplement: Figure S1 [file rsos172404supp1.docx]

**ESM Fig S1**

**for Thevenon & Pfuhl: Discrepancies in the spiking threshold and frequency sensitivity of nocturnal moths explainable by biases in the canonical auditory stimulation method**

**
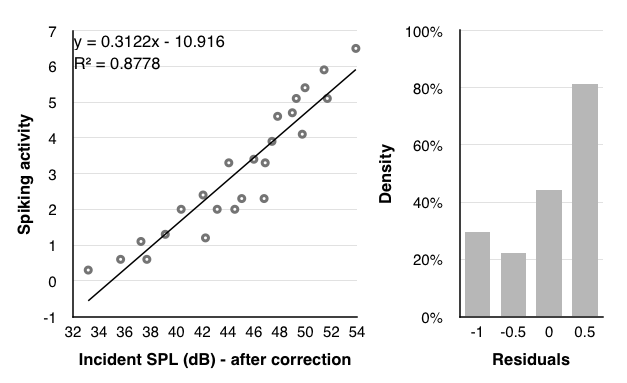
**

***Fig. S1. Linear regression applied to Fullard’s data corrected and evaluation of the residuals.***
