# Supplementary material for: Discrepancies in the spiking threshold and frequency sensitivity of nocturnal moths explainable by biases in the canonical auditory stimulation method
Source: R Soc Open Sci. 2018 Apr 11;5(4):172404. doi: 10.1098/rsos.172404 (PMC5936950; doi:10.1098/rsos.172404)
Supplement: Table S1 [file rsos172404supp2.docx]

**ESM for Thevenon & Pfuhl: Discrepancies in the spiking threshold and frequency sensitivity of nocturnal moths explainable by biases in the canonical auditory stimulation method**

**Table 1a. reported test parameters and SPL meter for spiking activity papers**

| 1. **Spiking activity papers** | **Pulse duration (ms)** | **Repetition rate (Hz)** | **SPL meter** | **SPL meter design** |
| --- | --- | --- | --- | --- |
| Waters, 1996 | 1.5, 3, 6.25, 12.5, 25, 50 | 1 | B&K2204 | 1969 |
| Surlykke et al., 2003 | 10 | 5 | ? | N/A |
| Göpfert & Wasserthal, 1999 | 30 | 10 | B&K2606 | 1969 |
| Fullard ,1984 | 3 | 1 | ? | N/A |
| Coro & Pérez, 1983 | 45 | 1 | ? | N/A |
| Fullard et al, 1998 | 45 | ? | B&K2607 | 1970 |
| Boyan & Fullard, 1986 | 10 | 1 | B&K2606 | 1969 |

**Table 1b.** **reported test parameters and SPL meter for spiking threshold papers**

| 1. **Spiking threshold papers** | **Pulse duration (ms)** | **Repetition rate (Hz)** | **SPL meter** | **SPL meter design** |
| --- | --- | --- | --- | --- |
| Waters & Jones, 1996 | 10 | 1 | B&K2204 | 1969 |
| Hofstede et al., 2011 | 20 | 5 | ? | N/A |
| Madsen & Miller, 1986 | 10 | 10 | B&K2606 | 1969 |
| Surlykke et al., 2003 | 10 | 1 | ? | N/A |
| Surlykke et al., 2003 | 5 to 30 | 1 | ? | N/A |
| Skals & Surlykke, 2000 | 50 | ? | B&K2607 | 1970 |
| Rydell, Skals, Surlykke, & Svensson, 1997 | 10 | 1 | B&K2606 | 1969 |
| Jackson, Asi, & Fullard, 2010 | 20 | 1 | B&K2610 | <1980 |
| Göpfert & Wasserthal, 1999 | 30 | 3 | B&K2331 | ? |

? means the information was not found in the published paper
