# Supplementary material for: Discrepancies in the spiking threshold and frequency sensitivity of nocturnal moths explainable by biases in the canonical auditory stimulation method
Source: R Soc Open Sci. 2018 Apr 11;5(4):172404. doi: 10.1098/rsos.172404 (PMC5936950; doi:10.1098/rsos.172404)
Supplement: Table S2 [file rsos172404supp3.docx]

**ESM for Thevenon & Pfuhl: Discrepancies in the spiking threshold and frequency sensitivity of nocturnal moths explainable by biases in the canonical auditory stimulation method**

**Table S2a. Data related to incidence corrections in the papers focused on spiking activity**

| **Spiking activity papers** | **Sound source** | **Microphone** | **Grid on/off** | **Microphone incidence to sound source** |
| --- | --- | --- | --- | --- |
| Waters, 1996 | n/a | B&K 4135 | off | n/a |
| Surlykke et al., 2003 | TH400B | GRAS 40BF | off | n/a |
| Göpfert & Wasserthal, 1999 | TH400B | B&K 4135 | off | n/a |
| Fullard, 1984 | KEF T27 | B&K 4135 | n/a | n/a |
| Coro & Pérez, 1983 | n/a | n/a | n/a | n/a |
| Fullard et al., 1998 | TH400B | B&K 4135 | n/a | n/a |
| Boyan & Fullard, 1986 | PH10 based design | B&K 4135 | n/a | “normal” |

**Table S2b. Data related to incidence corrections in the papers focused on spiking threshold**

| **Spiking threshold papers** | **Sound source** | **Microphone** | **Grid on/off** | **Microphone incidence to sound source** |
| --- | --- | --- | --- | --- |
| Waters & Jones, 1996 | N/A | B&K 4135 | off | n/a |
| ter Hofstede et al., 2011 | ScanSpeak 60102 | B&K 4138 | n/a | n/a |
| Madsen & Miller, 1987 | Bespoke design | B&K 4138 | n/a | “150 degrees” |
| Surlykke et al., 2003 | TH400B | GRAS 40BF | off | n/a |
| Surlykke et al., 2003 | TH400B | B&K 4135 | n/a | n/a |
| Skals & Surlykke, 2000 | Bespoke design | B&K 4135 | off | n/a |
| Rydell et al., 1997 | TH400B | B&K 4135 | on | n/a |
| Jackson et al., 2010 | ScanSpeak  no model | B&K 4135 | n/a | n/a |
| Göpfert & Wasserthal, 1999 | TH400B | B&K 4135 | off | n/a |
